# Dance and the Embodied Social Cognition of Mating: Carlos Saura’s Tango in the Perspective of the Tie-Up Theory

**DOI:** 10.1007/s12124-025-09895-7

**Published:** 2025-02-18

**Authors:** Lorenza Lucchi Basili, Pier Luigi Sacco

**Affiliations:** 1 Gender Strategies Center, Chieti, Italy; 2 Department of Neuroscience, Imaging and Clinical Studies, University of Chieti-Pescara, Chieti, Italy; 3 metaLAB (at) Harvard, Cambridge MA, USA

**Keywords:** Tie-up theory, Embodied cognition, Romantic relationships, Dance, Social cognition, Fictional narratives

## Abstract

This paper analyzes Carlos Saura’s film *Tango* through the theoretical lens of the Tie-Up Theory to explore how fictional narratives can serve as laboratories for investigating the embodied social cognition of romantic relationships. The study shows how dance, particularly tango, functions both as subject matter and cognitive metaphor in representing the complex dynamics of couple formation and maintenance. The film’s meta-representational structure, combining the creation of a dance performance with the exploration of actual relationships, reveals how cultural forms serve as cognitive scaffolds for understanding complex social dynamics. The study contributes to our understanding of how artistic representation can reveal typically implicit aspects of relationship cognition by demonstrating the value of integrating multidisciplinary perspectives of cognitive theory, psychology of mating, and cultural theory.

## Introduction

Among the diverse manifestations of human movement, dance stands out for its unique ability to integrate physical, emotional, and social dimensions of experience (Bigand et al., 2024) into a complex yet unified form of expression (Karpati et al., 2015; Christensen et al., 2017). In particular, dance holds a distinct position in its intimate connection to mating processes (Quiroga Murcia et al., 2009; Byers et al., 2010; Grammer et al., 2011). Through partner dance, potential mates engage in a form of physical proximity that might otherwise be constrained or prohibited by prevailing cultural norms (Hanna, 2010). This observation extends beyond cultures with strict physical contact rules. Dance serves as a crucial medium of multidimensional interaction—physical-biological, kinesthetic, and emotional—enabling potential partners to deepen their understanding of each other through embodied social cognition mechanisms to which humans are inherently attuned (Ravn, 2017). The centrality of dance in mating dynamics is evidenced by its recurring role in fictional narratives about courtship and romance, exemplified by enduring tales such as Cinderella or Beauty and the Beast (Lucchi Basili & Sacco, 2018).

The reference to fictional narratives as a window into dance’s role in human mating is not arbitrary but grounded in cognitive science. While empirical research has provided extensive insights into the key factors influencing mate selection across both sexes (Buss & Schmitt, 2019), such studies face an inherent limitation: they primarily capture preferences related to idealized partner choices or artificial selection scenarios rather than ecological settings (Lucchi Basili & Sacco, 2020a). Therefore, laboratory-based findings, while valuable and internally consistent, may not fully reflect the complex interplay of factors that influence real-world mate selection decisions (Eastwick & Finkel, 2008). This limitation is particularly significant when considering how embodied cognition and emotional responses shape partner selection in naturalistic contexts (Kille et al., 2013).

The challenge of studying ecological mate selection choices—with their obvious ethical constraints about manipulation or controlled observation—finds a valuable, and possibly surprising, alternative empirical pathway through fictional narratives. This apparently paradoxical approach derives its validity from fiction’s demonstrated capacity to function as a sophisticated simulation with significant social cognition content (Elster, 1999), a phenomenon increasingly validated by cognitive psychology research (Oatley, 2016; Mar, 2018). While fictional narratives present imagined scenarios and characters (even when adapting historical events or figures), cognitive science has investigated fiction’s remarkable ability to serve as a social simulation laboratory, enhancing readers’ capacity to interpret and understand others’ intentions, desires, and beliefs in real-world contexts (Barnes, 2018; Kidd & Castano, 2013, 2019). These acquired socio-cognitive competencies carry significant adaptive value, evidenced by specific neurocognitive and neuroaffective responses to fictional immersion (Jacobs, 2015; Willems & Jacobs, 2016). Fiction thus functions as a cognitive scaffold that shapes and guides our daily experiential flow, often at subconscious levels. As Frank astutely observes, “people have often forgotten the stories that *think* in them” (Frank, 2010, p. 14, emphasis added).

In addition to fiction’s established role in social cognition, cognitive science has unveiled another crucial dimension: the deep connection between fictional narratives and embodied cognition (Kuzmicová, 2014; Fischer & Zwaan, 2009). Contemporary neuroimaging research documents how when we engage with fiction, our brains activate sensorimotor circuits in ways that mirror real-world physical experiences (Speer et al., 2009; Gallese & Guerra, 2012). This embodied engagement becomes particularly salient in narratives featuring explicit physical activities like dance. Such stories trigger multimodal simulations at the neural level, simultaneously engaging mental, emotional, and sensorimotor systems (Chow et al., 2014). The neural activation patterns observed during the processing of narratives suggest that readers/viewers partially simulate the physical experiences being described, developing a rich, multisensory engagement with the material (Meineck, 2018; Gallese, 2019).

When fictional narratives represent dance – especially in the context of courtship or established relationships -- they offer uniquely valuable apprehensions into the embodied dimensions of human mating behavior. The coupling of physical movement and interpersonal dynamics in dance narratives creates a particularly fruitful domain for studying how embodied social cognition operates in mate selection and relationship maintenance. Watching dance performances, no matter whether in live shows or in fictional settings, activates not only the neural circuits associated with movement observation and simulation (Calvo-Merino, 2010) including those of individuals with no physical dance experience (Jola et al., 2012), but likely also those involved in processing social signals and evaluating potential mates, in view of the well-documented effects of dance on social cohesion (Hagen & Bryant, 2003) and mate attractiveness (Wade et al., 2015). This multilayered activation helps explain why dance has been a persistent motif in courtship narratives across cultures and throughout history. By representing the physical vocabulary through which potential partners evaluate compatibility and establish synchrony, dance narratives provide an accessible window into typically implicit aspects of mate selection that are difficult to study through conventional experimental methods. The metaphorical resonance between dance partnership and romantic partnership is thus not merely a literary convention but reflects fundamental processes of embodied social cognition that shape how humans form and maintain romantic bonds.

The challenge lies in developing theoretical frameworks capable of systematically analyzing how these embodied dimensions of mating behavior, so vividly captured in dance narratives, relate to the broader dynamics of romantic relationship formation and maintenance. While cognitive science has demonstrated fiction’s capacity to simulate complex social processes, and neuroscience has revealed the deep connections between physical movement and social cognition, we still need conceptual tools that can integrate these insights into a coherent model of relationship dynamics. This integration is particularly crucial for understanding how embodied compatibility assessments interact with conscious partner preferences and social expectations in determining relationship outcomes.

The Tie-Up Theory, which has been developed in previous work (Lucchi Basili & Sacco, 2016, 2017), provides an integrative theoretical framework that helps reconcile the apparent tensions between experimental findings and ecological reality. While laboratory studies offer valuable evidence, they often struggle to capture the full complexity of mate selection in natural contexts (Lucchi Basili & Sacco, 2020a). Our theory addresses this limitation by analyzing fictional narratives as sophisticated simulations of ecologically valid mating choices, incorporating a full spectrum of cognitive-embodied dimensions of partner selection. This approach has proven particularly effective in understanding the social cognitive value of enduring romantic narratives – stories that achieve successful intergenerational transmission both locally and globally. The theory’s applicability has been tested across diverse narrative forms, from classic fairy tales (Lucchi Basili & Sacco, 2018) to Hollywood romantic comedies (Lucchi Basili & Sacco, 2017, 2019). Even new contemporary narrative trends such as (some) Korean romantic TV miniseries (K-dramas) can be analyzed as powerful social simulations through this theoretical lens (Lucchi Basili & Sacco, 2020b).

Not all romantic narratives serve as effective vehicles for social cognitive insight – indeed, most contemporary romantic fiction fades quickly from cultural memory due to its poor or nonexistent social cognition content. The stories that endure typically demonstrate what cognitive narratologists term “psychological realism” (Mar & Oatley, 2008; Mar et al., 2011) – the ability to credibly simulate the cognitive-emotional-embodied dimensions of relationship formation. Recent work in cognitive poetics (Jacobs, 2023) and neuroscientific approaches to literature (Armstrong, 2013) suggests that this psychological authenticity operates independently of surface plausibility. Stories can offer profound inside views into human mating processes even when their settings or circumstances appear implausible or unrealistic, provided they accurately capture the embodied and psychological dynamics of human relationships.

The social cognitive value of fiction – its ability to convey implicit insights into human mating dynamics – emerges through sophisticated neural mechanisms of character identification. Recent studies in cognitive neuroscience demonstrate that when we engage with fictional characters, we activate brain networks associated with both social cognition and motor simulation (Gallese & Guerra, 2019; Broom et al., 2021). This neural engagement enables readers and viewers to process fictional characters’ experiences with remarkable psychological depth, treating them as fully realized individuals whose experiences carry meaningful social information (Oatley, 1994; Smith, 1995). However, while this social cognitive value is necessary for a narrative’s cultural persistence, it alone is not sufficient. Some narratives with high social cognitive potential may remain unrecognized, though they can be rediscovered through cultural mechanisms of adaptive reuse (Spolsky, 2015). Conversely, romantic narratives lacking substantial social cognitive value rarely achieve lasting cultural significance, regardless of their immediate popular appeal.

Our study applies the conceptual framework of Tie-Up Theory to analyze a narrative that foregrounds embodied experience through dance, particularly examining its insight potential for romantic human relationships. We focus on Carlos Saura’s *Tango* movie, which offers an exceptionally rich exploration of embodied cognition in relationship formation and maintenance. Recent work in cognitive film theory suggests that cinema provides unique perspectives into embodied social cognition by integrating visual, auditory, and kinesthetic elements in ways that still activate viewers’ sensorimotor systems (Gallese & Guerra, 2019; Heimann et al., 2019). Tango dance, with its complex integration of physical movement, emotional expression, and interpersonal dynamics, serves as both subject and metaphor in Saura’s work, making it particularly valuable for our analysis.

The film transcends conventional romantic narrative tropes to explore fundamental human experiences from a male perspective: aging, vulnerability, and mortality (Tan, 2020). This approach aligns with what McCormack (2013) identifies as a Deweyan experiential framework, where artistic expression serves as a laboratory for investigating lived experience. The narrative structure – centered on the protagonist’s creation of a tango show – operates on multiple cognitive levels. First, it functions as historical commentary, using tango to interpret Argentine cultural history (Hontang, 2017). Second, it serves as a meditation on romantic relationships, explored through tango both as physical practice and as metaphor for emotional connection. This dual perspective gains additional complexity because both the protagonist’s former partner and potential new love interest are dancers in his show. The film thus creates a sophisticated meta-representational structure: the lived experience of relationships is filtered through dance, which is then filtered through theatrical representation, and finally through cinematic representation – each layer adding new dimensions of embodied and social meaning.

A Deweyan interpretative lens reveals how Saura’s *Tango* functions as an aesthetic laboratory for investigating human experience (Johnson, 2007). Within this framework, the film becomes a phenomenological space (Dewey, 1929) where problem-solving centers specifically on the male protagonist’s romantic dilemmas. The Tie-Up Theory provides the analytical framework for evaluating both the protagonist’s choices and the various possible resolutions – or non-resolutions – of his relational challenges. Of particular significance is Saura’s deliberate construction of two contrasting endings, presented in juxtaposition, with the ultimate interpretation left to the viewer. This artistic choice aligns with contemporary understanding in cognitive narratology that fictional simulations serve not to prescribe solutions but to enhance our capacity for understanding relationship dynamics through narrative worldmaking (Herman, 2009, 2013).

Neither the film nor the analysis through the lens of its social cognitive value aims to offer definitive solutions. Instead, they provide interpretive frameworks for understanding the multiplicity of possibilities inherent in any romantic relationship. This multiplicity mirrors tango itself, whose improvisational structure generates countless variations of interpersonal meaning within established patterns (Van Alphen, 2014). Recent research in embodied cognition suggests that such structured improvisation serves as a powerful metaphor for relationship dynamics, as both require continuous negotiation between established patterns and spontaneous adaptation (Sevdalis & Keller, 2011).

Our methodological framework thus approaches the film as a cognitive simulation laboratory, analyzing key narrative moments through the Tie-Up Theory’s conceptual tools. This enables us to treat the movie as a narrative experiment (Gough, 2008), whose results offer insights into both the fictional protagonist’s perspective and broader patterns of male relationship experience. This approach builds on established work in cognitive narratology (Zunshine, 2006, 2012), of particular value for understanding how individuals navigate complex emotional and relational challenges.

## Theoretical Background

In this section, we synthesize key findings from several interconnected research domains that inform our analysis: fiction’s role as a channel of social cognition, dance as a medium for embodied social cognition in romantic relationships, and tango’s specific significance as a laboratory for studying sexual role dynamics within couples. We conclude with a focused overview of Tie-Up Theory’s fundamental principles. These theoretical foundations provide the necessary framework for our analysis of *Tango*’s narrative simulation, which we present in the subsequent section.

### Fiction as a Channel of Social Cognition

A crucial yet often overlooked aspect of human social cognition is its fundamentally reciprocal nature. Contemporary cognitive science demonstrates that humans don’t simply construct mental models to understand others’ intentions, motivations, and beliefs – they actively shape others’ perceptions to enhance mutual comprehensibility. Neuroimaging and cognitive science studies concur in indicating fictional narratives as powerful tools for the coordination of human subjects within shared meaning frameworks that make intentions, motivations, and beliefs mutually intelligible (Mar, 2011; Dodell-Feder et al., 2013; Zawidzki, 2013; Black & Barnes, 2015). Central to these coordination processes are embodied mechanisms through which mental and emotional state alignment is facilitated by physical interaction and postural/movement synchronization (Gallotti et al., 2017) that feels natural and effortless rather than forced or calculated (Ackerman & Bargh, 2010). These processes generate forms of participatory sense-making (De Jaegher & Di Paolo, 2007) in which dance may play an important role (Hermans, 2022), and whose reciprocal social regulation proves particularly significant in couple interactions, especially during negotiation and conflict resolution (Edward, 1995; Korobov, 2023).

The embodied dimension of social cognition isn’t merely a byproduct of cognitive structuring through functional narratives and narrative self-construction. Rather, as demonstrated by neuroscientific research, it lays the foundation of these processes (Hustvedt, 2011; Gallagher, 2017). The discovery that the same neural circuits govern both directly perceived experiences and mentally simulated ones suggests that the boundary between actual experience and imagination is far more permeable than traditionally assumed (Gallese, 2011; Bergen, 2012). This understanding is further supported by research on the Default Mode Network – the neural system active during ‘rest’ states – which shows particular activation during both daydreaming, and narrative simulation and imagined social interaction (Starr, 2013; Andrews-Hanna et al., 2014). Positioning the narrative self in terms of embodiment connects to a neo-pragmatist perspective wherein problem-solving through environmental interaction necessitates the construction of a unified experiential domain that draws equally from physical reality and imaginative resources (Menary, 2008; Johnson, 2007).

Cinematic fiction offers particularly fertile ground for studying these embodied simulation processes. The unique combination of sensory immersion and physical stillness characteristic of film viewing enables audiences to fully deploy their simulation capabilities in response to portrayed events. Watching characters perform actions activates corresponding premotor areas in viewers’ brains, even while remaining physically still (Shaw, 2016; Gallese & Guerra, 2019). This creates an embodied simulation at a safe distance (Wojciehowski & Gallese, 2011), allowing viewers to fully experience the emotional and sensorimotor dimensions of characters’ experiences while maintaining the protective frame of aesthetic experience.

### Dance: Social Cognition and Meaning Creation through Movement

Dance represents a unique form of human expression where meaning creation through non-verbal channels achieves exceptional complexity and completeness (Dunagan, 2005; Krug, 2022). Contemporary cognitive science recognizes dance as a distinct pathway for social cognition with profound historical-cultural significance (Sevdalis & Keller, 2011; Bläsing et al., 2012; Charnavel, 2019). Dance practice generates several psychophysical and social benefits (Quiroga Murcia & Kreutz, 2012; Muro & Artero, 2017; Vecchi et al., 2022), which, in partner dances, increase proportionally with experience and frequency of engagement (Lakes et al., 2016). This makes the analysis of dance-centered fictional narratives doubly significant from a cognitive perspective: they engage both the social cognitive mechanisms associated with fiction processing and those linked to sensorimotor experience.

Dance serves particularly complex signaling functions in human mating processes, leveraging the rich metaphorical significance of expressive movement (Müller & Ladewig, 2013; Fink et al., 2021). In fact, dance communicates crucial characteristics of potential partners through sophisticated sensorimotor channels (Hugill et al., 2010; Richter & Ostovar, 2016). This signaling becomes especially significant in male-to-female communication, where dance can convey multiple fitness indicators. These include body symmetry and masculine physical features that particularly appeal to female observers (Fink et al., 2007), risk propensity and sensation-seeking as markers of physical vitality (Hugill et al., 2011), physical strength (Hufschmidt et al., 2015), and specific personality traits (Fink et al., 2012). Motion-capture studies show how these signals correlate with specific movement patterns (Neave et al., 2011) and can even reflect distinct genetic characteristics (Bachner-Melman et al., 2005).

Partner dance creates an additional, highly specific channel for embodied social cognition through the negotiation of movements between dancers, leading to varying degrees of coordination success (Flakne, 2019). Such coordination culminates in what phenomenological research describes as a shared experiential space (He & Ravn, 2018). Eye-tracking studies highlight how effective motor coordination in dance facilitates partner trait memorization through enhanced facial observation time (Woolhouse, 2014). Female interest in potential male partners manifests in couple-specific patterns of motor coordination (Grammer et al., 1998), and neural activity linked to social cognition shows heightened activation in the following partner compared to the leading one (Chauvigné et al., 2018). These findings suggest that partner dance serves as both a medium for, and metaphor of, relationship dynamics, offering a rich domain for studying embodied social cognition in romantic contexts.

### Tango

Among improvisational partner dances, tango holds particular significance due to its exceptional complexity, rich stylistic variety, and deep cultural resonance. Its global appeal reflects what anthropologists identify as robust cross-cultural patterns in embodied social interaction (Savigliano, 1995; Davis, 2015). Cognitive research analyses of tango practice show how it relies on a sophisticated balance between improvisation and predetermined structure (Ravn, 2016), enabling dancers to spontaneously generate hundreds of movement combinations from a basic repertoire within minutes. This requires a rich portfolio of sensorimotor micro-skills: subtle physical competencies that develop through sustained practice and partner interaction (Kimmel, 2015).

The coordination levels demanded by tango, particularly in its most virtuosic forms, often transform the couple into what cognitive scientists describe as a superindividual ensemble (Kimmel, 2012). This transformation enables the maintenance of fluid, coordinated movement through the development of a predictive sensorimotor system (Davidson & Wolpert, 2005; Biscarini, 2021). Such coordination reflects into distinct neural signatures that can be reconstructed through neuroimaging, and that are significantly more pronounced in experienced dancers compared to novices (Amoruso et al., 2014). This suggests that tango expertise represents a radically emergent outcome of complex dynamic processes (Kimmel et al., 2018). The dance’s therapeutic potential has also been documented, with studies demonstrating its effectiveness in maintaining motor abilities in individuals affected by neurodegenerative conditions such as Parkinson’s disease (Koh et al., 2018; Lötzke et al., 2015).

Like other forms of synchronized movement with profound expressive significance, tango generates forms of kinesthetic empathy among its practitioners (Strukus, 2011; Fischman, 2015). It has been documented that empathy levels among tango dancers consistently exceed those observed in other collective dances requiring less interpersonal coordination (Koehne et al., 2016). This enhanced empathic capacity may explain why organizational theorists increasingly invoke tango as a model for understanding complex social coordination in various contexts (Pattinson et al., 2020). The dance’s unique combination of structure and improvisation, together with its demands for constant attunement between partners, creates an extraordinary embodied laboratory for studying interpersonal dynamics (Kimmel & Preuschl, 2016).

### Tie-Up Theory

The Tie-Up Theory (Lucchi Basili & Sacco, 2016, 2017, 2020a) provides an innovative conceptual framework for analyzing heterosexual couple formation and maintenance. This approach offers a parsimonious account of seemingly contradictory findings in relationship research while integrating insights from evolutionary psychology, cognitive science, and social neuroscience. The theory models heterosexual interactions through two distinct functional areas within each male (M) and female (F) individual: the Active Area (AA) and the Receptive Area (RA). What distinguishes the AAs is their being appointed to action in a predominantly conscious modality, and their sensitivity to socio-environmental factors such as status, social conventions, approval, behavioral models and lifestyles, and so forth. The RA, instead, is appointed to reception, to the acquisition of information that is processed at a mostly subconscious level, and if not activated does not manifest consciously. The activation of the RA depends on the outcome of a specific Compatibility Test that assesses the suitability of potential partners.

A fundamental aspect of the theory is the mirrored dimorphism between male and female areas: in men, the M-AA exhibits a sexual orientation while the M-RA has a psycho-emotional orientation. Conversely, in women, the F-AA demonstrates a psycho-emotional orientation, while the F-RA exhibits a sexual orientation. This structural difference means that F-RA activation depends on the male subject passing a sexual (and ultimately biological) Compatibility Test, whereas M-RA activation relies on the female subject passing a psycho-emotional Compatibility Test. When a potential partner passes the relevant test, it activates the RA of the subject that carried it out, predisposing the testing subject toward recruiting their own AA to focus the interaction with the other subject upon the possible formation of a couple.

**Fig. 1:**
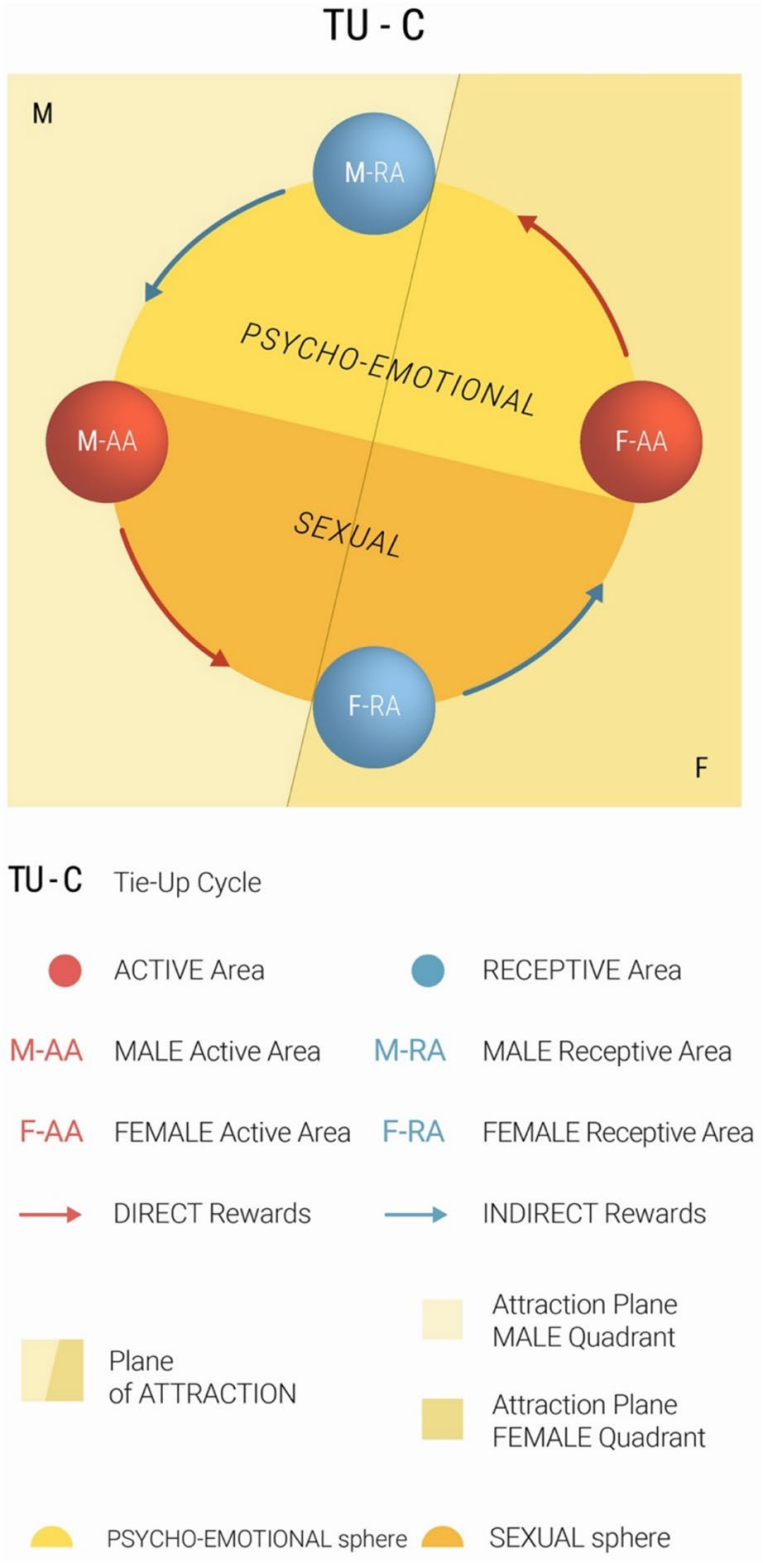
The tie-up cycle

The interaction structure is formalized in the Tie-Up Cycle (TU-C), which involves sequential stimulation of both subjects’ AAs and RAs in a characteristic order and cyclic direction. While the cycle can begin at any area, the starting point influences its progression and potential interruption. The sequence involves direct RA-to-AA communication within each subject, while AAs interact directly with the opposite subject’s RA. In an uninterrupted cycle, internal transmission flows from RA to AA within the same subject, followed by an external leap from one subject’s AA to the opposite subject’s RA. Communication between areas relies on specific rewards: AAs produce direct rewards, while RAs generate indirect rewards. A naturally flowing TU-C moves counterclockwise (Fig. 1), though this direction may partially reverse under certain conditions, such as when a subject frustrates rather than rewards the opposite subject’s RA.

The TU-C can be analyzed through two different categorical divisions: male/female or sexual/psycho-emotional. The male/female division groups areas as male (M-AA, M-RA) or female (F-AA, F-RA), while the sexual/psycho-emotional division groups them by orientation: sexual (M-AA, F-RA) or psycho-emotional (M-RA, F-AA). The evolutionary strategy underlying these connections facilitates interactions between opposite-sex subjects that, if repeatedly iterated, might lead to the formation of a stable, cooperative couple, whose bond would enhance joint parenting success. However, the iterations of the Cycle, and even less their interruption, provide no guarantee of such a positive outcome. An interesting parallel exists between the coordination required for maintaining an active TU-C through mutual gratification and the sensorimotor coordination between dance partners, making the latter a powerful cognitive metaphor for the former. As two badly matched dancers might disturb each other in their dance moves resulting in a dis-harmonic performance, two badly aligned partners in the production of their reciprocal rewards might likewise ride a malfunctioning Cycle, with iterates itself with difficulty.

As already remarked, partner dance carries an important social cognition content, with a double level of meaning, literal and metaphorical. Like partner dance, TU-C coordination has a pronounced embodied component, manifesting through complex nonverbal communication deeply rooted in sensorimotor dynamics. This embodied dimension extends beyond visual and auditory elements to include proprioceptive, tactile, olfactory, and gustatory signals, reaching even to the level of shared microbiota between partners (Kort et al., 2014; Montiel-Castro et al., 2013).

The TU-C can initiate even without either or both subjects having performed their respective Compatibility Tests. However, as cycle iterations progress, these tests naturally emerge through the exploratory production of indirect rewards by the respective areas. Upon successful completion of a Compatibility Test by the tested subject, the testing subject’s RA begins a sustained production of indirect reward. If, and when, such gratification reaches sufficient intensity, it establishes a Tie-Up (TU) in the testing subject’s RA with the successfully tested subject. This Tie-Up may be unilateral—either M-TU or F-TU, depending on whether it involves the male or female subject—or reciprocal, resulting in a Double Tie-Up (D-TU).

While a D-TU neither guarantees, nor is necessary for, couple formation (Lucchi Basili & Sacco, 2020a), it significantly influences relationship dynamics. When a D-TU forms and strengthens through subsequent TU-C iterations, the resulting couple demonstrates an improved resilience to environmental stressors and heightened probability of long-term stability.

Although the RAs determine Tie-Up formation through their activation, AAs play crucial roles in either amplifying or attenuating signals from their respective RAs (Lucchi Basili & Sacco, 2020a). For men, the activation of the M-RA cannot dispense with successfully checking the psycho-emotional compatibility of the female partner. For women, on the contrary, F-RA activation will be linked to the successful checking of the partner’s sexual and ultimately biological compatibility. This relationship is entirely mirror-like when considering AAs: the male AA consciously processes signals in the sexual domain, while the female AA consciously responds to psycho-emotional signals.

These conscious AA orientations typically emerge in studies eliciting ideal partner traits, which consistently show men prioritizing women’s physical attractiveness and women emphasizing men’s social and material resources (Buss & Schmitt, 2017). However, these abstractly desirable characteristics not only don’t guarantee the formation of a Tie-Up but might even preclude it. Real couples might form in the absence of Tie-Ups for both partners, based on sexual or social attraction alone, or could be matched to pursue instrumental societal incentives following the logic of the AAs. But such couples would remain vulnerable to dissolution if purely opportunistic. Moreover, excessive focus on direct rewards during the interaction that precedes the formation of a possible couple may prevent the possibility of a Tie-Up, if the corresponding RA never achieves sufficient activation to generate indirect rewards, thereby preventing the TU-C from fully iterating.

Thus, consciously expressed partner preferences do not fully span the actual mechanisms of Tie-Up formation, as they overlook some crucial but not always evident factors for the purpose of the Tie-Up, which operate primarily at subconscious levels. Relationship neuroscience studies support this distinction between conscious preferences and unconscious compatibility assessments (Cacioppo et al., 2012; Ortigue et al., 2010). As we have seen, contrary to common assumptions, sustained male bonding requires robust psycho-emotional compatibility (Wahring et al., 2024), while sustained female bonding similarly requires sexual and biological compatibility (Havlicek et al., 2008). In opportunistic couples, direct and indirect rewards can be strategically allocated between different partners: women would mate with resourceful male partners while looking for sexual and biological compatibility in extra-marital sex (Pillsworth & Haselton, 2006), whereas men would mate with psychologically compatible women while looking for recreational sex outside the couple (Labrecque & Whisman, 2017; Zhang et al., 2021).

Beyond Compatibility Tests, AAs also conduct specific Filter Tests on potential partners. Unlike Compatibility Tests, these Filter Tests are identical for both sexes (even if interpreted through the gender orientations and individual characteristics of the respective AAs) and reflect social and cultural criteria that vary across time and space. Primary Filter Tests evaluate psychological and biological traits, socio-economic status, and cultural factors. For instance, while female physical attractiveness may not establish compatibility for the male, it can serve as a Filter Test criterion, particularly when the male’s social position demands that his partner’s physical qualities function as status markers. Similarly, although a male’s psychological characteristics may not ensure female Tie-Up formation, women may still screen out potential partners with undesirable personality traits before carrying out their Compatibility Test.

When Filter Tests and Compatibility Tests positively or negatively align, the interaction improves or precludes the perspectives of formation of a couple, according to test results. However, when results diverge, subjects may experience internal conflicts, often exacerbated by external social pressures—for example, when a partner fails social adequacy Filter Tests despite passing the Compatibility Test. In such cases, outcomes become highly uncertain, depending largely on conflict resolution strategies, thus generating an extremely varied and complex spectrum of cases (Lucchi Basili & Sacco, 2020a). Couples may still form despite such conflicts but typically demonstrate increased fragility due to absent or unstable Tie-Ups, particularly compared to relationships founded on robust, non-conflictual D-TUs.

### Tie-Up Theory and Embodiment

The mechanism underlying the TU-C, based on the appropriate sequencing of direct and indirect rewards, naturally lends itself to interpretation through the lens of embodied cognition. Contemporary neuroscience suggests this connection operates on two levels: first, through the translation of reward vectors into specific patterns of neural activation, and second, through the fundamental role of sensorimotor processes in generating and transmitting the signals that produce both direct and indirect rewards. Neuroimaging studies seem to confirm that both physical and psychological compatibility assessments rely on complex information streams derived from posture, movement dynamics, facial micro-expressions, and the full spectrum of nonverbal communication (Cacioppo et al., 2017; Cacioppo, 2018), and that moreover relationship stability is also supported by neural mechanisms that implicitly derogate the attractiveness of potential alternative partners (Meyer et al., 2011).

This stream of neuroscientific evidence has fundamentally revised our understanding of romantic love, revealing it as far more than a mere, possibly transitory emotion, or altered state of consciousness. Instead, it emerges as a sophisticated cognitive and affective process linked to both subcortical and cortical reward pathways that promote goal-directed behaviors with significant adaptive value. For instance, studies of women bonded to opposite-sex partners (where the bond is defined as thinking about their partner more than 80% of their daily conscious mental time) show that subconscious exposure to the partner’s name—a signal directly addressing the RA—significantly enhances performance on cognitive tasks (Bianchi-Demicheli et al., 2006), possibly due to specific dopaminergic reward mechanisms. More broadly, subliminal partner-name priming activates neural networks associated with reward, pleasure, and higher cognitive functions (Cacioppo et al., 2012).

Reaction times for inferring the intentions of romantic partners consistently outperform those for non-romantic relationships (Ortigue et al., 2010), and moreover romantic partners elicit motor imitation significantly more than close platonic friends (Maister & Tsakiris, 2016), supporting the theory that embodiment plays a crucial role in couple-specific social cognition. This embodied dimension of social cognition relates to the reactivation of stored sensorimotor experiences during recognition processes. Such recall becomes increasingly automatic and less cognitively filtered as partner familiarity increases (Liew et al., 2011; Cacioppo et al., 2017). Experimental evidence about romantic fictional narratives further supports this embodied view of relationship cognition. In a series of studies, Gibbs (2013) demonstrated that people’s understanding of metaphorical narratives about relationships involves physical simulation. Participants to the experiment literally walked further when processing metaphors about relationships where success is translated as movement (“this relationship went very far”) compared to descriptions of relationship success which entailed no reference to movement. This suggests that our comprehension of relationship dynamics intrinsically involves embodied simulation processes rather than purely abstract conceptual understanding.

Within the framework of Tie-Up Theory, these findings can be understood as reflecting the increased salience of affect-laden nonverbal signals encoded during previous TU-C iterations and the subsequent development of a shared space of intersubjective representation (Alterman, 2007; Trevarthen, 2012). Through this shared space, couple interaction becomes conceptualized and perceived, ultimately leading to stable neural mirroring mechanisms (Ortigue & Bianchi-Demicheli, 2008). From this perspective, the effects of a fully operational TU-C as characterized by Tie-Up Theory show intriguing parallels with the Self-Expansion Model in romantic relationships (Aron et al., 2013), suggesting promising directions for future research.

While similar shared representational spaces can form in other close relationships like friendships, research indicates that these are more susceptible to disruption by external factors (Ruscher et al., 2003). Conversely, romantic relationship engagement can actually impair cognitive performance when tasks are performed without partner-related primes (Van Steenbergen et al., 2014), suggesting the depth of cognitive integration achieved through the TU-C process.

For emotional tasks such as recognizing others’ affective states, partner-related primes significantly enhance performance in bonded individuals. Notably, this improvement manifests particularly strongly in men’s ability to recognize negative emotions (Wlodarski & Dunbar, 2014), an observation that Tie-Up Theory would connect to the orientation of the M-RA. Similarly, research demonstrates that romantic relationships foster gradual overlap in bodily representations, making the embodied dimension a partially shared experiential domain (Quintard et al., 2021).

Engagement in TU-C produces significant embodied behavioral changes. For example, individuals in romantic relationships demonstrate enhanced inhibitory control over negative emotions compared to single individuals, with this effect particularly pronounced in recently formed relationships (Song et al., 2016). From the perspective of Tie-Up Theory, this improvement reflects behavior aimed at sustaining TU-C iteration through mutual reward production, including the regulation of subjective negative emotional states. While stable, satisfying marriages (or more generally stable long-term relationships) predict enhanced protection from negative well-being (Hsu & Barrett, 2020; Grundström et al., 2021) and demonstrate significant salutogenic effects (Horn et al., 2013; Robles et al., 2014), also by means of the enhanced promotion of healthy behaviors especially for men (Pylypchuk & Kirby, 2017), the emergence of psychological disconnection from one’s partner sharply reduces both psychological well-being and neuropsychological resilience (Cacioppo, 2018)—findings that powerfully illustrate both the benefits associated with a D-TU and the potential costs linked to TU-C interruption and dissolution.

## Carlos Saura’s *Tango* as a Laboratory for the Social Cognition of Intimate Relationships

We are now ready to analyze the results of our narrative experiment, based on the film *Tango* by Carlos Saura. This film represents a synthesis of the various elements introduced earlier: a fictional narrative centered on dance—and tango in particular—that allows us to examine the theme of the romantic couple from a male perspective within a representational context deeply rooted, both literally and symbolically, in sensorimotor aspects. We will focus on one of the film’s main dialogue scenes, which is particularly significant in the light of Tie-Up Theory, situating it within the broader narrative framework.

The interactional dynamics in this scene, which involves the male and female protagonists of the story, are characterized by a lack of ‘synchronization’ between the functioning of the male and female AAs and RAs. This establishes a natural cognitive parallel with the synchronization of dancers in a ballroom couple. The entire language of the conversation—interpretable as a flow of signaling between the protagonists’ functional areas, in ways that we will analyze below—clearly aligns with a representational metacognition strongly centered on embodiment.

This highlights how Saura’s choice to focus on tango is not merely a narrative device. Rather, it represents an exploration of human relationships through a particularly compelling lens. This choice allows for a rich, interconnected combination of verbal and non-verbal elements of communication and meaning. In the film, these elements are further accentuated through an overt sensory thematization, conveyed by the use of a different dominant color for each dance scene.

### Dancing Together does not mean Dancing as One

“I am sort of solitary animal… One of those old lions… who roam the African savannah aimlessly” (Saura, 1998, p. 15). This is what the male protagonist of the film declares while confessing his feelings to a young and attractive woman, a tango dancer who, due to her skill, beauty, and youth, has become one of the key figures in the tango show he is producing. It is interesting to note how his words represent a certain type of M-AA—namely an older, battered man, marked by the passage of time, a survivor of a serious car accident, convalescing and frustrated by the failure of his previous relationship due to the partner’s infidelity. The accident forces him to move with difficulty, using a cane—an ironic plight for a tango choreographer (and certainly skilled dancer) like him.

Comparing himself to an old solitary lion effectively describes a M-AA that feels sovereign, yet perceives that his power is waning due to age. For this reason, he tends to isolate himself, remaining within his territory—a loneliness that implicitly suggests, as the metaphor of the solitary old lion does, a loss of seductive leadership over what the dominant male considered his ‘female harem.’ A king who senses and fears the impending loss of strength and power. The savannah, the domain of all male AAs, is the Sexual Plane through which male identity is negotiated, which describes an ongoing need for dominance in various fields of interest and ambition.

All AAs exist to be active and take pleasure in being so. In the case of men, being active aligns with pride in their supremacy, power, and dominance. To act, achieve, and conquer means for a M-AA to be at the helm of his own kingdom, whatever it may be. The metaphor is thus quite fitting, exemplifying the specific gender identity. And what about women? Is it the same for them? The character in the film continues: “Lionesses are different. They gather, unite, hunt… whelp, nurse… protect their defenseless cubs… They have a concrete mission in life” (Saura, 1998, p. 15).

Here, the focus shifts away from dominance to an admiration that a male AA might express for the ability to engage in meaningful action, appreciating the fulfillment that comes from doing and being active. This list of actions is filled with wonder and approval, conveying sincere esteem. A perspective that represents the active viewpoint of masculinity towards femininity. Unlike him, these women do not wander aimlessly; they have “a concrete mission in life,” thus realizing their sovereignty without too much difficulty—simply by acting and giving meaningfulness to their existence. This saves them from solitude and the aimless search for a territory to exercise their hegemony. From an AA’s viewpoint, how could they not be pleased and satisfied with their immense power to act and create?

However, the young interlocutor does not seem to resonate with this: “That’s a woman mission in life?” (Saura, 1998, p. 15), leading him to respond, confused: “I didn’t mean that. I respect women too much. Maybe that was a bad analogy” (Saura, 1998, p. 15). The example was intentional and fitting, but it did not yield the hoped-for outcome: approval for the clarity of his analysis and a gratification for the recognition of the existential merits of the female gender. He sincerely expressed his viewpoint with the intent to seduce her, but what did he achieve? The opposite: the woman shows irritation, and the man does not fully understand why. However, wisely and cautiously, he apologizes, retracing his steps and admitting his mistake—even if he does not really comprehend what it was.

What did he do wrong? He confused the sphere of action of the F-AA with his own. The sphere of action of the AAs is not the same for the M-AA and the F-AA, and the man failed to recognize this, merely extending his sexual identity to the female identity sphere, quite glaringly missing the mark. The sphere in which any F-AA operates is not the sexual but the psycho-emotional one, meaning that mating, procreating, nursing, etc., concern the female sexual field, the F-RA, and do not necessarily involve the psycho-emotional aspect of the F-AA, for whom an emphasized sexual physicality and role modeling sounds more like being considered breeding and labor animals, devoid of dignity and competence, than actual individuals. From a M-AA’s viewpoint, this is akin to an unflattering comparison between a king lion of the savannah and a domesticated cow on purely utilitarian grounds.

The man attempts to explain himself again, shifting the metaphor from animals—the lion—to action—the hunt. Action is what fundamentally interests any AA: the sensorimotor sphere is their natural playing field. In this way, he tries to articulate the foundations of his male identity while hoping to pinpoint and resolve the root of his current frustration: “Men… have been raised to hunt and fight… for thousands of years. Now… he hunts in his own way. Let’s say he goes haywire… for a little power… or a medal or money, a form of power. It’d be a shame if women… followed men in their folly. That’s what I meant” (Saura, 1998, pp. 15–16).

The desire for dominance is reiterated. Hunting, any kind of hunting, unequivocally expresses a favorite activity of M-AAs, as it is the ultimate symbolic expression of achieving one’s goals. Snatching the prey represents an opportunity for dominance, the power to decide over a being/object that, if it does not manage to escape, attests to an ability, a trophy that serves as “a medal,” providing full satisfaction. A gratification marked by the achievement of merit long sought after by the M-AA, which thus certifies and circumscribes, in identity terms, its power to act.

However, the protagonist has been cheated on by his woman, who left him for another man, and this has spiced up the challenge to his identity as a hunter. Thus, the man’s frustration leads him to reflect that it would be a true shame if women were to compete by “imitating men’s folly” even in this regard.

His regret, when read from the vantage point of the sphere of action of the M-AA, indicates a concern that women, in their desire to imitate men, forsake their receptive stance, and thus their passive role, in the sexual plane of the TU-C. The term “imitate” indeed refers to adopting the same active modality within the same realm of action—the sexual identity sphere—thus obviously mixing up the cards on the table. We are thus confronted with an intriguing tangle of misunderstandings. Focusing only on dominance yields a form of psychological fragility that prevents a clear understanding of the nature of the problems that have arisen, which ultimately ended up coalescing around a single disappointment regarding the loss of power inherent in aging, which deeply tarnishes the identity dimension. For this reason, managing to seduce a different, attractive woman—also younger than the previous one—would resolve all frustration, and replenish vigor and confidence. However, the protagonist, also due to the accident that forces him to use a cane, realizes that he now belongs to a different tribe, that of the not-so-young, thus doubting a direct approach to the F-RA of the girl on a sexual level, and instead trying to convince her F-AA with a sound logical and rational argument regarding the psycho-emotional sphere. And it is here that he begins to get confused.

First mistake: failing to acknowledge the change of sphere. The move to change the sphere of interaction is correct, especially because it brings considerable strategic advantages. Seducing the F-AA is a first appropriate step, as it allows for preparing the ground for a subsequent capture of the F-RA, in order to arrive at the female Tie-Up, the sincere infatuation of the woman, which the man hopes, one last time, to seize for himself. But if he approaches an F-AA expecting to maintain the perspective of the M-AA within the sexual sphere, as a sort of defensive bastion of an identity that constantly fears being challenged, then he certainly risks either offending or, at the very least, failing to enchant the F-AA. Following this route leads to denying her perspective, which is entirely different, and for a reason. An Active Area that lies within a diametrically opposed plane of the Tie-Up Cycle, and thus has different needs. The approach thus turns out to be extremely crude and insensitive, one might say even dumb and, most importantly, uninterested in considering a way of viewing things other than his own. For women, this indicates a lack of consideration and respect, even when the man has no such intention. He appears as someone boasting of his arrogance, showing no interest in the other, ignoring or denying any viewpoint other than his own. However, in our case, it’s simple ignorance. This male character simply fails to properly address an Active Area—the F-AA—and also ignores that this area belongs to the psycho-emotional plane of the TU-C, with different needs from those of his own Active Area, the M-AA.

Here comes the second mistake: confusing the F-AA with the F-RA, failing to distinguish their differing nature. Continuing to think in terms of the sexual sphere, he believes he is addressing a Receptive Area that is passive, as in this plane of the TU-C it is he who is active. This explains why he generally accuses F-AAs of willing to “imitate” male behavior, which he believes is the only active modality that would ever exist. Like a tango dancer who leads the dance but ignores that being ‘active’ is intrinsic and inherent to any F-AA. Tango offers us a poignant analogy for the context of the film: while the male partner in a tango couple is active on the interpretation of the music, deciding how to “read” it and kinesthetically translating it through his own personal style of dance, which establishes the elegance of the whole performance, the female partner is indeed apparently passive in adapting to this interpretation, but she is also active in the sphere that highlights her most, that of the dance embellishment, of crafting her own steps that transform the performance into a sensual and visually complex experience. This intersection of active modalities, when coordinated masterfully, allows for a mutual enhancement of the performance of the dancers. It is as if, in the tango couple, the male and female steps denote the harmonic and melodic structures of the musical piece respectively, and it is above all the melody that captures the listener’s attention (and similarly, in the case of tango, it is primarily the female steps that attract the spectators’ gazes).

The scene in the film showcasing the young dancer performing solo, accompanied only by the orchestral musicians, emphasizes, somewhat curiously, the female steps in a tango with an absent male partner, symbolizing a dance in which the passive female role has been omitted in favor of an exclusively active female one. The movement appears limited if compared to a partner dance, perhaps because the dancer focuses more on her embellishments than on the choreutic translation of the musical rhythm.

It is not, therefore, necessarily an “imitation.” The male and female protagonists are differently active because their AAs belong to different planes of the TU-C. So how does a M-AA differ from a F-AA? If for the former dominance is the biggest gratification of identity, for the latter dominance would represent only a revenge without leading to the achievement of a gratification with an identity value. This is explained very clearly by the female character in the film, who responds to the man as follows: “All I ask of men… is to respect me, listen to me… and not treat me like a nut spouting nonsense. Why is it so hard for men to admit that a beautiful woman… can also be intelligent? Must a good mind always go with an ugly face?” (Saura, 1998, p. 16). The F-AA claims her own sphere of action in the psycho-emotional plane where her intelligence is recognized, along with all her countless applications in terms, for example, of sensitivity, intuition, and analytical-deductive thinking, without having to relinquish her femininity and sexuality for this—something that is far removed from any pretension of imitation.

The female viewpoint dismisses any need for dominance in favor of emphasizing unity and holistic completeness, without excluding any parts, almost underscoring the necessity of psychological attention that extends to the whole individual and from them to other individuals. Attention is shifted from dominance to the relationship, envisioning a more egalitarian approach even in leadership.

Yet our man is too ensnared in his frustrations to understand, responding, “That’s not what I think! Look!” (Saura, 1998, p. 16), striking a narcissistic pose, in fact not only ignoring but also toppling the call for consideration onto a sphere from which men shy away, because in the psycho-emotional plane of the TU-C their relative position would be passive, being represented by the M-RA. He is completely consumed by his abstinence from direct rewards that his M-AA is not producing for him. He must only overcome the crisis, resolve his problem of failure: The woman serves this purpose, he does not need her thoughts. Thus, he attempts again, trying to explain directly: “How can I put this? You wake up one day, look in a mirror… and say: ‘I’ve aged.’ You go outside, and the young call you mister. They look at you askance. To them, you’ve gone over the hill. You’re an old fart. Time goes by… your hair starts to fall out… then the rest falls apart. You like good food, you get fat… you get lazy, stop going out. Still… despite the physical decay… you feel as energetic as a boy. So what do you do? Why is it unseemly for a man to act like a boy? I can’t enjoy a girl of 18 because I’ an older man. How old are you, anyway?” (Saura, 1998, p. 16). She smiles and radiates as if she appreciates the question finally, responding, “Tventy-three,” and he, “You seem younger,” almost disappointed for some reason, and hastily continues, “Let’s see if I can complete this. On that day you wonder… ‘What life have I had? What’s happened to me? Where are my youthful illusions, my dreams?’” (Saura, 1998, p. 17).

There are no answers to these questions in the film, which instead of telling a single version of the story, rather presents the viewer with a series of hypotheses and possibilities, like in a collection of tango texts, culminating in two distinct endings—first the tragic one, where jealousy, vengeance, and death triumph, then the joyous one, where love emerges and the new couple is formed. The relentless passage of time and the flow of life might lead the protagonist to fear that he has missed the opportunity to delve deeper: “I only touched the surface of things. All I did was to swim frantically… to avoid sinking into the muck. How much have I swum!” (Saura, 1998, p. 17). Or it might prompt him to self-reproach harshly: “It’s all wrong. You got carried away by your imagination. That’s not enough, and you know it. You need more discipline.” More discipline, in what? In love? In dance? In life? “What’s the dramatic line that unifies all this?”. The man switches to his role as the director (nodding at the meta-narration): “The woman leaves him. The guy falls apart.” In his own previous words: he no longer swims. “But another woman appears. A younger dancer. They fall in love,” and immediately, as the director, he wonders, “- He falls in love? Does he fall in love? Or is he grasping at straws?” (Saura, 1998, p. 17). Here again, the possibilities are twofold.

The Tie-Up Theory would posit that it all depends on which male Area has become involved. If it involves only the M-AA, then yes, falling in love represents a lifeline, an overdose of direct rewards that will help him bounce back, to “get back to swimming” boosted with energy and self-esteem. If, however, we are talking about the M-RA and indirect rewards, then falling in love is serious, the kind of story where one no longer dances tango when their dance partner is gone, because their lifelong companion has died. This is what happens to another character in the story, an elderly man teaching tango to the youngest, but who hasn’t danced since he became widowed. “And her? Does she fall in love?” he continues to ponder, answering himself, “She exploits it.” The doubt remains. He admits, “I don’t know.” According to the Tie-Up Theory, a woman can also fall in love, let’s say, with two different levels of attachment, depending on whether only the F-AA is involved, or whether the F-RA is also seduced. The film seems to opt for the utilitarian version, one that emphasizes the personal advantage for the young woman in catering to the powerful man, despite his age, in exchange for quick professional advancement or, for instance, for protection from the previous exploited man, whom she has left and whose reaction she fears. In this case, the man chosen by the F-AA is a source of solid direct rewards, even if fleeting. “He pursues her, despairs if she’s not there. She’s everything to him. She hides, slips away. She plays.” Yet if the sexual act also generates indirect rewards for the F-RA, the woman will genuinely fall in love, since this second type of gratification is self-sustaining, and the connection could solidify if the couple’s interaction is good and the TU-C gets on track with its flow of both types of rewards. Nonetheless, whichever hypothesis is correct, it matters little. Only time would tell, and if time runs short because the man feels he is aging, “He has no time for games”, then he might well consider himself lucky that this young and beautiful woman, whether in love or not, has chosen him for whatever reason.

Then the director returns to question his previous relationship, asking himself, “What will the woman do? She returns?” and he answers himself, “She returns!” so that a triangle can form, and tango can be danced even among three. But then he imagines what she might say to him: “I love you. I adore you. I need you.” and immediately comments with a sigh, “What a lot of nonsense. Never changes.” Will it be a real F-TU or merely a simulated one? He is again floating on the surface, and his sentimental frustration could re-emerge. And thus, another unresolved inquiry arises. Deliberately left open to allow for this possibility: “But cannot the story be told differently?”

A different way, for example, would be to narrate the story making use of the resources offered by the TU-C. One would then realize that many of the words the protagonist uses gain additional significance. Let’s try. He laments remaining “on the surface of things,” and indeed that is what he does with the girl when, seeking to propose himself to her, opening up and explaining himself, he misses his opportunity to delve deeper into his interest in her, and perhaps even to fascinate her genuinely. He recounts himself with sincerity and a keen analytical ability, but he can read only himself. He is the center of his own interest while she is merely a means to feel better, to feel a bit younger again. This is what it means staying on the surface in a relationship. Swimming, and staying afloat “to avoid sinking in the muck,” he says, to feel good again thanks to the direct rewards the woman will assure him by offering herself and remaining by his side. Until when? Until the rewards run out again. “How much I have swum!” insinuates evident fatigue. Yes, this type of reward resembles a drug to which one quickly becomes addicted, and the loss of effect is inevitable. His constant surface-level existence is reflected in the failure of his previous relationship. His partner left him. Why? He merely thinks the reason is that she preferred another man. He likely does not question this simple conviction because the center is always him and his wounded ego. How could we describe this superficiality in the TU-C?

If a man satisfies himself solely with his direct rewards, he remains trapped in the sexual plane, and in this way cannot leverage the propelling force of the Tie-Up Cycle, which, like an engine, would allow him to “stop swimming” without stopping his motion, that is to say, without losing his rewards, and sinking. But why does one remain trapped in a plane? Because of two reasons that should always go together: because one is unable to engage both the female Active and Receptive Areas of the woman they are interested in, and because one does not pay enough attention to securing a sufficient share of one’s own indirect rewards. This amounts to failing to move toward the plane that lies on the opposite side to the sexual one, namely the psycho-emotional plane. The tango metaphor is once again useful. The tango master in the film advises: “You must feel the girl! I want to see one body with four legs! Let’s see some joy!” (Saura, 1998, p. 9).

The protagonist, in his romantic relationships, behaves as a tango dancer who does not feel his dance partner because he is too focused on his own steps to also care about the other dancer’s ones – expecting of her, as an expert, to follow along and support his idea of dancing. But she does not enjoy dancing that way and thus seeks another partner. The TU-C functions like a couple of dancers making the decision to dance together. For the dance to be enjoyable for both the viewer and the dancers, the connection must be excellent, and the interaction needs to be correct. There should be no imbalances, nor excessive protagonism that would cause unpleasant disharmonies for everyone. To dance tango well, one cannot then “give in to imagination” because “imagination is not enough! You need more discipline.” In the Tie-Up Cycle, the man pursuing his direct rewards, those that only satisfy him in his sexual identity, behaves like the dancer who indulges in imagination while neglecting inspiration. “Inspiration, where are you?” the protagonist asks. He answers himself, “What is inspiration but work, work, work…” (Saura, 1998, p. 18).

The inspiration within the couple is found by the man in the woman, just as the dancer finds it in his dance partner. That’s where one should look. But to do this, the dancer must “feel” his partner and become one body with her, just as the man must get to know the woman by following her in the psycho-emotional plane, as Alice follows the White Rabbit into its hole. This means going deep, in dance as in a relationship, that is to travel through the TU-C repeatedly. What is the difference between completing the Cycle versus not completing it? The distinction lies in the indirect rewards and in the fact that also the M-RA now becomes excited. Obviously, this implies additional work for the man to achieve his own Tie-Up. It is a considerable commitment for the dancer, who must ensure that his interpretation of the music takes the characteristics of his dance partner into account so that the execution is harmonized, with the right fluidity of movement.

“Work, work, and work…” Because only work leads to the knowledge, which we might label awareness, from which inspiration springs. For this reason, the film begins and builds from dancers who practice their acts day after day in every corner of the immense stage, which could easily symbolize the various iterations of a Tie-Up Cycle necessary to establish D-TUs and reach cruising speed, when bodies become one body, and the TU-C begins to iterate by itself. The effort then becomes minimal, even nonexistent, in view of the amazing result achieved, for instance, in a dance performance that leaves everyone speechless.

But why do indirect gratifications, produced by the excitement of the M-RAs, make a difference? Because they do not exhaust like direct rewards, and when placed within a TU-C, they allow it to iterate effortlessly, overcoming any fatigue, as when dancers seem to move lightly at the peak of their performance, ready to soar. And above all, because experiencing indirect rewards confirms to the man the existence of a psychological compatibility with a specific woman, as when the tango dancer finds his ideal dance partner, with the right height, physique, affinity, personality, energy. Being passive in the psycho-emotional plane of the Cycle makes the man highly receptive to the psychological and emotional stimuli that a compatible woman can transmit to him, much more than if he were active, and so too occupied with doing, and too little with listening and processing.

Thus, recognizing a woman as compatible in the psycho-emotional plane, that is, the job of the M-RA, can become so exciting that it lends all the necessary inspiration for any creation. Discipline is therefore found in practicing and practicing, developing the sensitivity and connection required to dance with a woman. And in a relationship, discipline must ensure to avoid neglecting one plane of the Cycle to indulge only in the opposite one because travelling along the whole TU-C would imply interacting both sexually and psycho-emotionally, and consequently having to take into account also the otherness of their partner. The reward for this hard work cannot be really appreciated unless one has had some experience of the indirect rewards, at least once. It’s like pretending to imagine passion without ever having experienced it, and only passion is what drives and sustains tango dancers. Thus, age no longer matters, both in relationships and in artistic expression, and masters of tango, dancers, and musicians can tango even at eighty years, because their inspiration remains immune to the passage of time.

### Finding a Common Tempo in Dance and in Romantic Relationships: An Embodied Perspective

The conversation analyzed in the previous section provides an eloquent illustration of how embodied cognition operates in romantic interactions through the metaphorical framework of dance. This parallel becomes particularly significant when we consider how tango, as a partner dance, requires continuous negotiation of physical and psychological space between dancers (Tateo, 2014; Kimmel, 2015). Just as tango dancers must maintain a delicate balance between leading and following while preserving their individual expression, the conversation between the protagonists reveals a similar, demanding dynamic of attempted coordination and misalignment through trial-and-error.

The male protagonist’s metaphorical framework of the “solitary lion” reveals his embodied understanding of relationships through dominance and territory, a typical example of how abstract concepts can be embodied in physical experience (Gibbs, 2014). This framing stands in stark contrast to the cooperative nature of tango, where success depends on mutual attunement and we-agency rather than individual prowess (Forlé, 2024). The disconnect between his conceptual framework and the actual dynamics of romantic interaction parallels with the fundamental challenge in tango: the need to transcend individual technical competence to achieve genuine partnership (Kimmel & Preuschl, 2016; Kimmel and Van Alphen, 2022).

The conversation’s failure to achieve emotional resonance can be understood from the vantage point of participatory sense-making (Cuffari et al., 2015). Whereas tango requires partners to create a shared space of meaning through coordinated movement, successful romantic interaction points to the same goal through mutual alignment along the iterations of the Tie-Up Cycle. The protagonist’s inability to shift from his dominance-based framework to one of mutual coordination mirrors the common challenge of novice tango dancers who struggle to move beyond technical execution to achieve genuine connection with their partners (Zubarik, 2013; Ravn, 2016).

This parallel becomes particularly evident in the way the male character attempts to control the conversation’s direction through logical argumentation, much like an inexperienced tango lead who relies on predetermined sequences rather than responding to their partner’s feedback. As Flakne (2019) notes in her analysis of partner dance, genuine interaction requires a willingness to relinquish control and enter a state of mutual responsiveness. The protagonist’s rigid adherence to his perspective prevents the emergence of a shared experiential space essential for meaningful connection (Olszewski, 2008).

The embodied nature of this disconnect is further emphasized by the character’s physical condition—his need for a cane serving as a literal manifestation of his inability to engage in the fluid give-and-take that characterizes both successful tango and romantic interaction. This physical limitation clearly becomes metaphorically significant from an embodied cognition viewpoint, offering a glaring example of how an understanding of abstract concepts may be powerfully supported by suitable associations with physical experience (Gallese & Lakoff, 2005).

The female character’s resistance to the male protagonist’s metaphorical framework can be understood as a rejection of the “leader-follower paradigm” in its most basic form (Kimmel, 2019; Kimmel & Hristova, 2021). Her insistence on being recognized for her intelligence rather than her biological role parallels the evolution in tango from rigid gender roles to a more nuanced understanding of partnership, where following is recognized as an active rather than passive role (Davis, 2015).

This tension between different modes of interaction reflects broader patterns in how embodied cognition shapes our understanding of relationships. Successful social interaction requires the development of shared bodily representations that facilitate mutual understanding. The failure to achieve this in the conversation mirrors the physical disconnect that occurs when tango partners fail to establish a coordination pattern (Kimmel et al., 2018).

The embodied metaphor of dance becomes particularly powerful when we consider how both tango and romantic relationships require partners to develop predictive models of each other’s responses (Reddish et al., 2013; Cacioppo et al., 2017). Experienced tango partners learn to anticipate each other’s movements based on subtle physical cues, and this creates an intriguing parallel with stable romantic partners developing sophisticated models of each other’s characteristics and inclinations. The conversation’s failure to achieve such a level of attunement suggests a fundamental misalignment in these predictive models.

Likewise, the protagonist’s difficulty in adapting his perspective mirrors the challenge of developing action simulation capabilities in dance (Cross et al., 2006; Sevdalis & Keller, 2011). A dancer must learn to simulate and anticipate their partner’s intentions (Grafton, 2009); accordingly, romantic partners must develop the ability to simulate and understand each other’s emotional and physical states. The character’s inability to move beyond his own framework flags his failure in this crucial aspect of social cognition.

The parallel between dance and relationship dynamics extends to the role of interconnectedness in creating meaning through movement (Bannon & Holt, 2012; Hermans, 2022). Tango requires partners to create meaning through their physical interaction, as well as romantic relationships demand the co-creation of a meaningful flow of reciprocal rewards along the Cycle. The conversation’s failure to achieve this level of co-creation reflects a fundamental misalignment in how the characters approach this process of meaning-making.

The embodiment perspective then enables us to appreciate how the conversation analyzed above simultaneously falls short in terms of relationship development on multiple grounds, each of which points to a clear metaphorical sensorimotor equivalent in couple dancing. This analysis suggests that the embodied metaphor of dance provides a powerful framework for understanding the dynamics of romantic interaction. The parallel between physical coordination in dance and psychobiological coordination in relationships highlights how embodied cognition shapes our understanding and experience of the connection to a partner. Future research should further explore how the embodiment framework and its metaphorical extension could inform our understanding of relationship dynamics and contribute to the development of more effective approaches to relationship counseling and education.

## Discussion: The Embodied Dimension of Romantic Relationships within the Tie-Up Theory Framework

The analysis presented in the previous sections highlights how the Tie-Up Theory provides a useful conceptual framework for understanding the embodied nature of romantic relationships and mate selection processes.

One of the core reasons for examining Carlos Saura’s *Tango* through the prism of the Tie-Up Theory, rather than any other artistic expression or film, is the way the dance form itself captures both the physical and psychological dimensions of mate selection and relationship dynamics. Whereas many other creative works offer abstract narratives of love and partnership, tango explicitly requires synchronized bodily movement and ongoing negotiation of personal space, making it an exceptionally powerful metaphor for the embodied processes described in the Tie-Up Theory. The dance medium materializes intangible elements of attraction (for instance, subtle physical and emotional cues, and mutual attunement) more vividly than most other art forms because its core aesthetic lies in balancing freedom of expression with structured interdependence. This duality parallels the interplay between conscious partner preferences and embodied signals that the theory proposes.

Additionally, Saura’s film provides a meta-representational framework, in which fictional dance performances become a laboratory for real-world relational tensions. This layered depiction—embedding choreography within a love triangle—showcases how artistic contexts may illuminate hidden facets of romantic interactions. While, as shown elsewhere (Lucchi Basili & Sacco, 2017, 2019, 2020b) other types of dances or films might also serve to illustrate important aspects of the Tie-Up Theory, tango’s improvisational potential and emphasis on lead-follow dynamics pinpoint the subtle interplay of conscious and subconscious mate-assessment processes with particular clarity. Thus, although the Tie-Up Theory would still apply to non-tango narratives, the film’s focus on dance-as-relationship offers a distinctive case that effectively highlights the uniquely embodied, culturally resonant aspects of human mating behaviors.

A key insight emerging from this theoretical perspective is how the sexually dimorphic structure of AAs and RAs reflects fundamentally different ways in which men and women experience and process embodied signals in romantic contexts. For men, the psycho-emotional orientation of the M-RA means that psychological compatibility is assessed through embodied interactions that generate indirect rewards, while their sexually-oriented M-AA responds to direct sexual rewards. Women’s complementary structure, with a sexually-oriented F-RA and psycho-emotionally oriented F-AA, creates a different pattern of embodied assessment and reward processing (Lucchi Basili & Sacco, 2016).

This dimorphic architecture helps explain why abstract partner preferences often fail to predict actual mate choices. In terms of the methodological concerns raised by Eastwick et al. (2014), stated preferences measured in experimental settings may primarily reflect AA-level criteria that operate consciously, while actual attraction and bonding depend heavily on RA-level compatibility assessments that occur through embodied interaction. The theory suggests that these RA-level processes rely on sophisticated neural mechanisms for processing multisensory social signals, as demonstrated by research showing how romantic love activates specific subcortical and cortical pathways (Fisher et al., 2005; Cacioppo et al., 2012).

The Tie-Up Cycle (TU-C) provides a dynamic model for how embodied interactions between partners can lead to stable bonding through the coordinated, reciprocal exchange of both direct and indirect rewards. This aligns with research on how physical synchrony and motor coordination in couples contribute to relationship satisfaction and stability (Gallotti et al., 2017). The theory’s emphasis on the need for both types of rewards maps onto findings about how successful relationships require both physical/sexual and emotional/psychological intimacy (Shaver et al., 2010).

Moreover, the theory’s distinction between Compatibility Tests and Filter Tests offers insight into why embodied attraction may conflict with consciously held preferences or social expectations. While Filter Tests operate primarily at the socio-cognitive level and are influenced by cultural norms, Compatibility Tests involve subconscious processing of embodied signals that may generate a powerful attraction regardless of whether a potential partner meets predetermined social criteria (Lucchi Basili & Sacco, 2020a).

The theory also helps explain the profound impact of embodied experiences on relationship dynamics. The process of forming a double tie-up (D-TU) involves complex patterns of reward generation and processing that engage cognitive-embodied systems. This aligns with research showing how stable romantic bonds involve integration of multiple neural systems, including those governing reward, motivation, and social cognition (Ortigue et al., 2010).

Particularly relevant is how the theory conceptualizes the role of indirect rewards in building stable relationships. The fact that these rewards typically operate below conscious awareness but can powerfully influence behavior aligns with research on implicit social cognition in relationships (Murray & Holmes, 1999). The theory suggests that these processes rely heavily on embodied signals that may not be accessible to conscious introspection but nevertheless shape relationship satisfaction and stability to a crucial extent.

Future research needs to investigate and characterize the neural mechanisms underlying the theory’s proposed AA/RA architecture, to examine how different types of partner-related stimuli activate distinct brain networks. Additionally, longitudinal studies could test the theory’s predictions about how patterns of embodied interaction influence relationship development and stability over time.

The theory also has potential practical applications for relationship counseling by highlighting the importance of attending to both conscious preferences and subconscious, embodied aspects of partner attraction and bonding. Understanding how these systems interact could help couples better navigate conflicts between rational relationship goals and embodied experiences of attraction and compatibility.

To sum up, the Tie-Up Theory provides a full-fledged framework for understanding how embodied processes shape romantic relationships. By integrating insights from neuroscience, evolutionary psychology, and relationship research, it may offer valuable perspectives on why mate selection and relationship maintenance often defy rational analysis and conscious intention.

## Conclusions: Fiction as a Laboratory for the Understanding of Romantic Relationships

Our analysis of Carlos Saura’s *Tango* through the lens of Tie-Up Theory demonstrates how certain fictional narratives can serve as sophisticated laboratories for investigating the embodied social cognition of romantic relationships. The film’s exploration of relationships through the metaphor of dance provides particularly rich insights into how physical and psychological dynamics intertwine in the formation and maintenance of romantic bonds.

The parallel between a tango partnership and a romantic relationship dynamics proves especially illuminating when analyzed through the framework of Tie-Up Theory. Just as successful tango dancing requires both technical competence and deep interpersonal attunement, stable romantic relationships depend on the coordination of both conscious and subconscious processes. The film’s depiction of the protagonist’s struggles—simultaneously an artistic director and potential romantic partner—reveals how these processes can become misaligned, leading to relationship instability.

Saura’s decision to present multiple possible endings reflects the complex, probabilistic nature of relationship outcomes predicted by the Tie-Up Theory and instantiated by the Mating Stability Matrix (Lucchi Basili & Sacco, 2020a). The film suggests, in alignment with the Tie-Up Theory, that relationship trajectories depend not just on conscious intentions or social circumstances, but on the complex interplay between embodied compatibility and conscious filtering processes. This aligns with contemporary understanding in cognitive narratology that fictional simulations serve not to prescribe solutions but to enhance our capacity for understanding relationship dynamics through narrative worldmaking.

The focus on tango as both subject matter and metaphor proves particularly effective for exploring the embodied dimension of romantic relationships. The dance sequences visualize how partners must negotiate physical and psychological space, reflecting the theory’s emphasis on how relationship stability depends on successfully coordinating both direct and indirect rewards. The film’s exploration of how age and physical limitation affect this dynamic—through the protagonist’s injury and dependency—provides insight into how embodied constraints can impact relationship formation and maintenance.

Moreover, the film’s meta-representational structure—showing the creation of a tango performance while exploring actual relationships—demonstrates how artistic expression can serve as a cognitive scaffold for understanding complex social dynamics. This once again stresses how engagement with fiction activates neural networks associated with both social cognition and motor simulation (Gallese & Guerra, 2019). Artistic creations with a strong embodiment focus may therefore contribute to our analysis of complex real-world relationship dynamics, providing powerful metaphorical tools that greatly enhance our understanding through the transposition of challenging representational problems onto cognitively more familiar grounds.

The study of fictional narratives through frameworks like the Tie-Up Theory offers significant advantages for relationship research. While experimental studies necessarily abstract from the full complexity of real-world relationships, fiction can present densely contextualized scenarios that capture the interplay of physical, psychological, and social factors. Fictional narratives allow us to explore how embodied and psychological aspects of relationships interact in ways that would be difficult to study directly in experimental settings. The use of artistic metaphors, such as dance, can make typically implicit aspects of relationship dynamics visible, particularly regarding their embodied cognition content. Through multiple narrative possibilities, fiction can illuminate how different combinations of compatibility and filtering processes lead to varying relationship outcomes. Furthermore, meta-representational structures in fiction can help reveal how cultural forms provide key metaphorical resources for understanding the complex, path-dependent nature of relationships. However, more cross-cultural research is needed to assess to what extent such structures possess universal vs. culturally-specific characteristics.

In conclusion, our analysis contributes to the stream of literature that highlights how fictional narratives, particularly those that foreground embodied experience like Saura’s *Tango*, can serve as valuable resources for understanding the complex dynamics of romantic relationships. The combination of artistic representation and theoretical frameworks like Tie-Up Theory offers fresh insight into how physical, psychological, and social factors interact in relationship formation and maintenance. This approach complements traditional experimental research by providing multi-layered, contextualized scenarios that could inspire more comprehensive theoretical accounts to better capture the challenging, intriguing variety of human romantic relationships.

## Data Availability

No datasets were generated or analysed during the current study.
